# Mucosal immunity to poliovirus

**DOI:** 10.1038/s41385-021-00428-0

**Published:** 2021-07-08

**Authors:** Ruth I. Connor, Elizabeth B. Brickley, Wendy F. Wieland-Alter, Margaret E. Ackerman, Joshua A. Weiner, John F. Modlin, Ananda S. Bandyopadhyay, Peter F. Wright

**Affiliations:** 1 Department of Pediatrics, Dartmouth-Hitchcock Medical Center, Lebanon, NH USA; 2 Department of Microbiology and Immunology, Geisel School of Medicine, Dartmouth College, Hanover, NH USA; 3 Department of Infectious Disease Epidemiology, London School of Hygiene & Tropical Medicine, London, UK; 4 Thayer School of Engineering, Dartmouth College, Hanover, NH USA; 5 Bill & Melinda Gates Foundation, Seattle, WA USA

## Abstract

A cornerstone of the global initiative to eradicate polio is the widespread use of live and inactivated poliovirus vaccines in extensive public health campaigns designed to prevent the development of paralytic disease and interrupt transmission of the virus. Central to these efforts is the goal of inducing mucosal immunity able to limit virus replication in the intestine. Recent clinical trials have evaluated new combined regimens of poliovirus vaccines, and demonstrated clear differences in their ability to restrict virus shedding in stool after oral challenge with live virus. Analyses of mucosal immunity accompanying these trials support a critical role for enteric neutralizing IgA in limiting the magnitude and duration of virus shedding. This review summarizes key findings in vaccine-induced intestinal immunity to poliovirus in infants, older children, and adults. The impact of immunization on development and maintenance of protective immunity to poliovirus and the implications for global eradication are discussed.

## Introduction

Successful implementation of the polio endgame will depend on immunization strategies that not only prevent paralytic disease, but also limit enteric replication of the virus and minimize the risk of transmission of potentially neurovirulent strains^1–4^. Vaccines containing live attenuated poliovirus have been the mainstay of immunization campaigns for decades, and remain a critical component of efforts to eradicate wild-type virus^5^. Oral polio vaccine (OPV) replicates extensively in the gastrointestinal tract and is distinguished by its ability to induce intestinal immunity, and decrease virus shedding upon subsequent exposure of individuals to live virus^6^. In contrast, inactivated polio vaccine (IPV), while inducing strong systemic immunity and protection from paralytic disease, has little effect on virus replication in the intestine following an OPV challenge in subjects previously not exposed to live virus^7^.

Concerns over the safety of OPV due to rare cases of vaccine-associated paralytic polio (VAPP) caused primarily by the type 2 component of the vaccine, and the introduction of vaccine-derived polioviruses (VDPV) into areas of low population immunity, led to the removal of the type 2 component from trivalent OPV (tOPV) and a globally synchronized switch from tOPV to bOPV (types 1 and 3) in 2016^8^. Coupled with this change was the recommendation to include at least one dose of IPV in immunization strategies to prime individuals against potential exposure to type 2 poliovirus, and to boost population immunity against all three serotypes^3^.

Vaccination regimens consisting of IPV, bOPV, and combinations thereof have since been evaluated in clinical trials to assess rates of seroconversion and virus shedding in response to a monovalent OPV (mOPV) challenge^9–15^. These trials revealed a marked difference in the degree of protection induced by tOPV versus bOPV and IPV against strain-specific enteric poliovirus replication as measured by recovery of type 2 virus in stool. To gain insight into the ability of combined bOPV and IPV regimens to induce intestinal immunity, mucosal antibody responses have been studied within the context of these trials^15–18^. Consistent with early observations, these studies demonstrate an important role for neutralizing antibody responses mediated by IgA in limiting replication of poliovirus in the intestine ^6,19–23^.

This review summarizes current findings in mucosal immunity to poliovirus and provides a theoretical framework for interpreting observed differences in vaccine-induced intestinal immunity to bOPV and IPV regimens across age groups. The broader implications for the prevention of poliovirus infection and transmission within global eradication efforts are discussed.

## Mucosal immunity to live and inactivated Poliovirus vaccines in infants

### Combined OPV and IPV as a primary series

Recommendations to assess combined OPV and IPV schedules in infants were first presented in 1987 by the Global Advisory Group of the WHO Expanded Program on Immunization^24^. An overarching goal for combined vaccine use was to improve early protection against polio and strengthen immunity in children from developing countries, where OPV-only schedules were associated with lower than expected rates of seroconversion^25–29^. Combining OPV and IPV to broaden immunogenicity to poliovirus was subsequently evaluated in a clinical trial of 1685 infants from The Gambia, Oman, and Thailand^30^. A seminal finding from this study was that IPV alone failed to provide adequate protection from poliovirus shedding, while the combined regimen of OPV with IPV induced strong seroconversion and intestinal immunity as measured by a reduction in virus excreted in stool.

Results from this and subsequent clinical trials^31–37^ formed the cornerstone of current efforts to evaluate the effectiveness of combined vaccine regimens in infants using new schedules of bOPV and IPV^9–15^. A central objective of these trials is to define the best strategy not only for preventing paralytic disease, but also for inducing intestinal immunity and limiting virus shedding. Using updated immunological techniques to measure polio-specific enteric antibody responses, further studies have linked the induction of intestinal immunity to the type and timing of the primary vaccine series^16–18^. When evaluated alongside concurrent data on virus shedding, these immunologic analyses have allowed a more nuanced picture to emerge of the induction and development of enteric antibody responses to bOPV and IPV regimens, and the relationship of these responses to virus shedding upon subsequent challenge with mOPV.

### Comparative studies of intestinal immunity to combined vaccine schedules

**Fig. 1:**
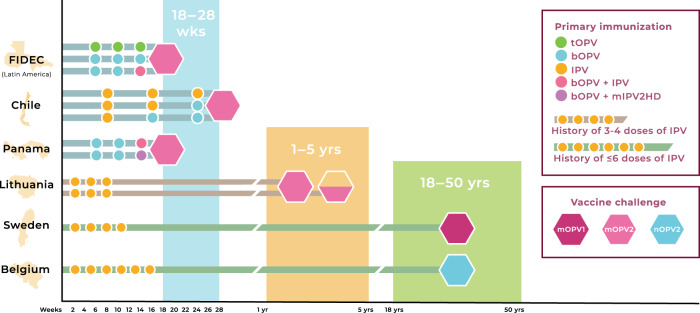
Global clinical trials of poliovirus vaccines in infants, children and adults. Clinical trials were conducted to assess induction of polio type-specific immunity in response to immunization and OPV challenge in infants (FIDEC, Chile, Panama)^11–13^, children (Lithuania)^51^, and adults (Sweden, Belgium)^54,56^. Induction of polio type-specific intestinal immunity for each trial was evaluated in a related set of studies^16–18,51,55,57^. Details of the primary immunization schedules and type-specific OPV challenges for each study are published and briefly summarized here. FIDEC (Fighting Infectious Diseases in Emerging Countries): Infants were randomized to receive a primary series of tOPV-tOPV-tOPV, bOPV-bOPV-bOPV or bOPV-bOPV-bOPV+IPV at 6, 10, and 14 weeks of age, followed by mOPV2 challenge at 18 weeks^13^. Chile: Infants were randomized to receive IPV-IPV-IPV, IPV-IPV-bOPV, or IPV-bOPV-bOPV at 8, 16, and 24 weeks of age, followed by mOPV2 challenge at 28 weeks^11^. Panama: Infants received bOPV at 6, 10, and 14 weeks of age and were randomized to receive a standard dose of IPV or a monovalent high-dose type 2-specific IPV (mIPV2HD) at 14 weeks, followed by mOPV2 at 18 weeks^12^. Lithuania: Children (ages 1–5 years) who had received 3–4 doses of IPV at 2, 4, 6, and 18 months of age were challenged with a dose of mOPV2 at study entry. A second dose of mOPV2 was given 28 days after the first in a subset of the children^51^. Sweden: Adults who had received 3–4 doses of IPV in childhood were challenged at study entry with mOPV1^54^. Belgium: Adults who had received up to six doses of IPV in childhood were challenged with a single dose of nOPV2 at study entry^56^.

Initial studies examined the induction of intestinal immunity to bOPV and IPV-containing regimens in comparison to the “gold standard” of a tOPV regimen. Infants from Latin American countries within the FIDEC (Fighting Infectious Diseases in Emerging Countries) consortium were studied to evaluate the immunogenicity of tOPV in comparison to two schedules of bOPV and IPV^13^ (Fig. 1). Not unexpectedly, robust intestinal IgA and strain-specific neutralizing responses were found in infants exposed to live poliovirus as a tOPV primary series^16^. These responses were evident on the day of challenge and corresponded to a marked restriction in type-specific virus shedding after oral exposure to mOPV2^16^.

In comparison, infants receiving a bOPV or bOPV–IPV series had negligible polio type 2-specific enteric antibody on the day of challenge. Significant type 2-specific IgA and neutralizing activity developed only after challenge with mOPV2 and reached peak levels comparable to those in the tOPV group by 2 weeks^16^. Addition of IPV to the bOPV series had no significant effect on virus shedding after challenge as compared with bOPV only schedules^16^. These results point to a restriction of type-specific virus shedding that is clearly associated with prior receipt of homotypic live virus (tOPV), and are consistent with early observations that induction of mucosal immunity to poliovirus in the intestine is stimulated by enteric replication of the virus^19,38^. Further, these results demonstrate the limited impact of a single dose of IPV when added after bOPV in a primary series to reduce fecal shedding following oral exposure to live poliovirus.

### Contribution of inactivated poliovirus vaccine to intestinal immunity

The impact of IPV on induction of intestinal immunity and efforts to elucidate the contribution of IPV in combined schedules with bOPV have been evaluated in clinical trials; however, these results can be confounded by prior receipt of live vaccine or by environmental exposures to OPV-related polioviruses^30,39,40^. A 2015 clinical trial conducted in Chile examined the immunogenicity of an IPV-only primary series, in comparison to IPV-bOPV schedules, in infants for whom there was no prior polio vaccine exposure^11^ (Fig. 1). High rates of seroconversion were found for infants receiving an IPV or IPV + bOPV series, yet further analysis showed only minimal enteric antibody responses prior to challenge in infants receiving the IPV-only series^17^. Challenge with mOPV2 induced a rapid intestinal antibody response in the IPV-immunized infants, characterized by a significant increase in polio type-specific IgA and virus neutralizing activity in stool 2 weeks after oral exposure to live virus. These findings again reinforce the concept that induction of type-specific enteric antibody to poliovirus in infants is stimulated by replication of live virus in the intestine. Moreover, in vaccine-naïve subjects, receipt of an IPV-only primary series is insufficient to induce significant levels of enteric IgA and virus neutralizing activity in the absence of OPV.

The conclusion that a primary IPV-only series does not induce sufficient intestinal immunity to reduce fecal excretion of poliovirus after oral live-virus challenge is consistent with evidence from several early studies^7,19,41^, and further aligns with a 2012 meta-review demonstrating the limited impact of IPV on fecal shedding within one month after challenge with live virus^42^. In light of these findings, our current understanding of the capacity of IPV to elicit mucosal immunity capable of reducing virus replication in the intestine, and the nature of the vaccine series needed to achieve this goal, remain uncertain^43^.

### Boosting intestinal immunity after priming with OPV

While it is clear that at standard doses IPV alone fails to stimulate mucosal antibody responses in the intestine, increases in mucosal immunity have been noted in studies in which a single dose of IPV was given to individuals previously primed by OPV^7,31,43–45^. The mechanism by which IPV boosts intestinal immunity in this context is not well understood but presumably reflects reactivation of polio-specific memory responses established during initial enteric exposure to live virus^7^. The magnitude of the boosting effect of IPV on OPV-primed intestinal immunity is most evident in older children and young adults, as compared to infants, and likely reflects waning of the intestinal response in the intervening period between oral exposure to live virus and the IPV boost^7,31,43,45^.

This finding raises questions as to whether the boosting effect of IPV on mucosal immunity might be enhanced in a primary series by increasing the dose of inactivated vaccine given in combined regimens with bOPV. A recent clinical trial conducted in Panama examined the gain, if any, in primary intestinal immunogenicity in infants receiving bOPV and either a standard dose of IPV or a novel monovalent high-dose type 2-specific IPV (mIPV2HD)^12^ (Fig. 1). Significantly higher rates of seroconversion and higher type-specific serum titers were associated with mIPV2HD as compared to standard IPV suggesting that increasing the dose of IPV may be effective for enhancing polio-specific serum antibody responses^12^.

In comparison, no clear benefit was found for high-dose IPV in stimulating intestinal immunity^18^. Polio type-specific enteric IgA and neutralizing responses developed similarly in both vaccine groups within 2 weeks of mOPV2 challenge and restriction of virus shedding was not substantively changed in infants receiving mIPV2HD as compared to standard IPV^18^. These findings underscore the distinction in immunological responses to IPV between the serum and mucosal compartments^19^, and further suggest these differences may not be effectively overcome by increasing the dose of IPV used in a primary schedule with bOPV.

Taken together these studies provide compelling evidence that enhancing serum neutralization through the addition of standard, or high-dose IPV to a primary bOPV immunization series does not yield a comparable gain in intestinal immunity upon subsequent exposure to live type 2 poliovirus. Moreover, these findings support prior observations that polio-specific enteric immunity is distinct from the serum response and is a primary determinant of virus shedding^6,19,23^.

Further insight into the intestinal antibody response emerged from the Panamanian study, which assessed polio type-specific IgA1, IgA2, IgG, IgD, and IgM levels in stool relative to virus neutralization and shedding following mOPV2 challenge^18^. While poliovirus type 2-specific IgD was inversely associated with viral shedding at 1 week after challenge, this response waned by 2 weeks and a stronger inverse correlation emerged between virus shedding and type 2-specific IgA. Among subjects shedding virus, levels of type 2-specific IgA1 correlated more strongly than IgA2 with both total IgA and stool neutralization, indicating that IgA1 may be the predominant isotype mediating mucosal neutralization of poliovirus in the intestine.

Collectively, this series of clinical and immunologic studies in Latin American infants demonstrates induction of polio type-specific enteric IgA and virus neutralizing activity on mOPV2 challenge in infants receiving either bOPV or bOPV–IPV as a primary series. A highly significant inverse correlation was found between the enteric mucosal responses and the amount of virus shed after challenge^16–18^. A central message emerging from these trials in infants is that prior live vaccine (tOPV or bOPV) induces strain-specific intestinal immunity and effectively limits virus replication on oral challenge^16^. Further addition of IPV (either standard or high dose) to a bOPV primary series has limited impact on the induction of enteric antibodies or virus shedding. This observation falls within the scope of a recent meta-analysis of serum and mucosal responses to different poliovirus vaccine regimens, which found no evidence of increased intestinal immunity to type 2 virus associated with the addition of IPV to a bOPV schedule^46^.

## Mucosal immunity in older children and adults

### Waning of vaccine-induced intestinal immunity to poliovirus

Fewer studies have examined mucosal immunity to poliovirus in older children and adults for whom the interval between the primary vaccine series and subsequent exposure to live virus may be considerably longer. Analyses of fecal IgA responses in older children show poor responses among those immunized with OPV in infancy, and challenged with live virus 9 years later as compared to infants vaccinated with OPV twice within an interval of 6 weeks^47^. The observation that revaccination with OPV after an extended period is associated with immunologic boosting supports the concept that intestinal immunity wanes over time^47,48^. Indeed, studies by Grassly and colleagues have shown that protection against virus replication in children receiving OPV partially wanes within a year of initial vaccination, and may be incomplete even after multiple vaccine doses^49^.

That intestinal immunity to poliovirus diminishes with age is supported by age-related studies in older children (1–4 years) showing that the odds of virus excretion increase significantly with the time since last vaccine exposure^49^. Studies by Jafari et al.^44^ show that fecal shedding of poliovirus in children with prior IPV occurs in a significantly higher proportion of older children (10 years of age) as compared to those 6 and 11 months at the time of bOPV challenge. Persistent virus shedding on OPV challenge has also been shown in an early study of adolescent volunteers with prior exposure to IPV or OPV in childhood^50^. Efforts at boosting intestinal immunity with a single dose of IPV appear to be more effective in children primed with OPV on average 7 months prior to the IPV boost^31^ as compared to serial administration of OPV and IPV as a primary series^16,18,30,33^, providing further evidence that intestinal immunity wanes after primary immunization and may be boosted by a subsequent later exposure to IPV^31^.

Together these findings support the concept that intestinal immunity to live poliovirus may either fail to develop, or progressively diminish over time in older children and adolescents leading to an increased likelihood of virus excretion upon oral re-exposure to live virus. This suggests that in the absence of additional vaccine doses, mucosal immunity capable of restricting poliovirus replication in the intestine diminishes after the primary childhood series, creating an immunological gap in older children, adolescents and adults.

### Age-related deficit in poliovirus vaccine-induced intestinal immunity

Recent studies have examined the development of intestinal antibody responses to poliovirus vaccines in somewhat older children. In a clinical trial conducted in Lithuania, children (ages 1–5 years) who had received up to four doses of IPV in accordance with the recommended schedule for pediatric immunizations were challenged with a dose of mOPV2 at study entry^51^ (Fig. 1). Despite evidence of strong polio type-specific serum neutralizing responses, only about one-third of children evaluated for mucosal antibody responses in stool achieved polio type-2 specific neutralization titers ≥32 after the first challenge dose of mOPV2. A similar percentage achieved a stool neutralization titer of ≥32 after a second challenge dose. Notably, high titers of virus were detected in a least one stool sample from a majority of children, who shed virus after the first or second challenge dose.

This observation in IPV-immunized children is in contrast to responses seen in vaccine-naïve children, for whom virus shedding is markedly diminished both in percent shedding and in mean titer shed with the second dose of the same strain of OPV^52,53^. Although these comparisons involve different populations of differing ages, one conclusion that can be reached is that initial receipt of IPV in infancy may lead to greater shedding after an OPV challenge in older children. This has profound programmatic implications for polio eradication, and may also be an important insight into the nature of immune control of poliovirus replication in the intestine. While well-immunized IPV populations have controlled poliomyelitis and, for the most part poliovirus transmission, evidence of increased virus shedding in IPV-immunized older children following oral exposure to live virus may be significant in terms of potential enhancement of silent wild-type or VDPV circulation, particularly in geographic areas of low vaccine coverage.

The relative absence of detectable enteric antibody responses to live poliovirus in IPV-immunized children as young as 1–5 years of age is consistent with suboptimal induction of intestinal immunity in this age range. As compared to findings in IPV-immunized infants, in which mucosal antibody responses in stool increased rapidly following OPV challenge^17^, these results in older children suggest an age-related defect in enteric mucosal immunity to poliovirus associated with an increased interval between immunization and live virus challenge.

### Absence of detectable intestinal IgA responses to OPV in young adults

The possibility that induction of mucosal immunity to poliovirus is less robust after infancy is further supported by recent findings from a study of Swedish adults, who had received IPV in childhood as part of routine immunization programs^54,55^ (Fig. 1). Not surprisingly, prior to challenge, subjects had no detectable poliovirus neutralizing activity in stool and undetectable enteric IgA to all three poliovirus serotypes^55^. Following mOPV1 challenge, all subjects experienced high titered and sustained virus shedding in stool. Unexpectedly, none developed significant enteric IgA responses to poliovirus during a 7-week follow-up after challenge. Neutralizing activity against two other commonly circulating respiratory viruses, influenza A/H1N1 and respiratory syncytial virus, was detectable in stool from the majority of the subjects arguing against a more generalized deficit in mucosal immunity.

Similar results were found in a recent study of Belgian adults, who had received IPV in childhood and were challenged with a single dose of a novel OPV type 2 (nOPV2) at study entry^56^ (Fig. 1). Despite rising serum neutralization responses, the majority of subjects failed to develop virus neutralizing titers in stool above the limit of detection (<4) and those with detectable responses achieved only very low titers^57^. These findings are consistent with an earlier report demonstrating rapid induction of systemic antibody responses but prolonged virus excretion extending up to 8 weeks after OPV challenge in elderly Dutch adults, suggesting the absence of mucosal immunity able to limit virus replication in the intestine^58^.

### Comparison of polio-specific intestinal antibody responses across age groups

**Fig. 2:**
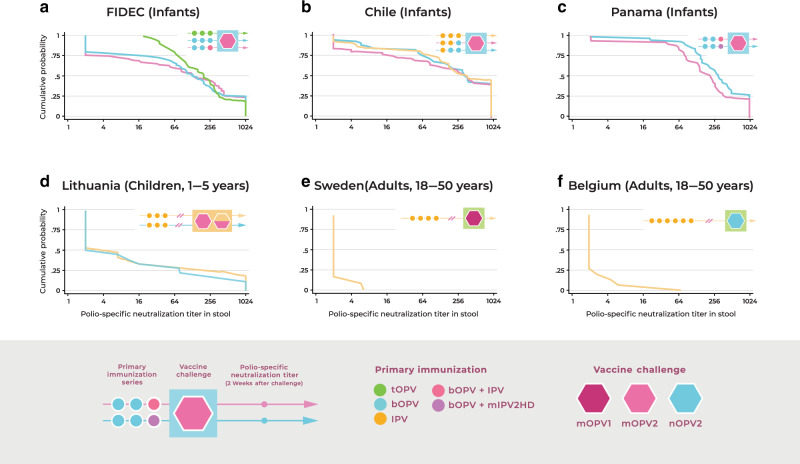
Age-related decline in intestinal neutralizing antibody responses to poliovirus following challenge with OPV. Polio type-specific neutralizing responses were measured in stool following OPV challenge in a series of studies of infants, children, and adults participating in clinical trials of poliovirus vaccines^16–18,51,55,57^. Data represent the reverse cumulative distribution of polio type-specific neutralizing titers measured in stool 2 weeks after OPV challenge in each study. The magnitude of the enteric response is shown as the cumulative probability of participants achieving a neutralization titer within the specified range. Comparative responses are shown for infants challenged with mOPV2 at 18–28 weeks (**A**–**C**); children challenged with mOPV2 at 1–5 years (**D**) and adults challenged with mOPV1 (**E**) or nOPV2 (**F**) at 18–50 years. Polio type-specific stool neutralization titers were measured as described^16–18,51,55,57^ and all samples were tested in the same laboratory using a standardized neutralization assay. Data for each study was analyzed independently to generate reverse cumulative distribution curves. FIDEC, Fighting Infectious Diseases in Emerging Countries.

In summarizing data on intestinal immunity from global clinical trials of bOPV and IPV regimens (Fig. 1) clear distinctions emerge in the induction of enteric type-specific neutralizing responses to poliovirus associated with different vaccine regimens and across age groups (Fig. 2). Two weeks after challenge with mOPV2, the pattern of neutralizing antibody responses measured in stool indicates a robust intestinal response to live-attenuated poliovirus occurs in infants (Fig. 2A–C). Typically strong type-specific enteric neutralizing responses are seen in infants receiving a primary series of tOPV (Fig. 2A). Neutralizing titers in stool are also readily detected in infants receiving a bOPV series (Fig. 2A) or a combined regimen of bOPV and IPV (Fig. 2A–C). Similar analysis of data from older children in Lithuania (Fig. 2D) and young adults in Sweden and Belgium (Fig. 2E, F), however, reveals a striking age-related diminution in the magnitude of the enteric polio-specific neutralizing response following OPV challenge.

When considered together, these findings from multiple clinical trials suggest that, unlike infants, older children and adults may be less able to mount mucosal IgA responses to poliovirus in the intestine, despite enteric replication of the virus and strong type-specific serum antibody boosts. This observation raises questions as to whether induction of intestinal immunity to poliovirus may be impaired or absent in adults, a finding that is in marked contrast to current evidence from infant studies^16–18^.

### Factors affecting levels of enteric IgA to poliovirus measured in stool

The lack of detectable enteric antibody responses to OPV in older children and adults may be attributed to a number of possible factors, including study-related differences in sample size, collection methods, or changes in the performance of laboratory assays used to quantify mucosal immunity. In several recent studies, assessment of enteric responses is substantiated by inclusion of data from two unique laboratory assays that quantify distinct immune variables (type-specific mucosal IgA and virus neutralization) and that are highly correlated with each other^16–18^, making it less likely that variation in laboratory methods accounts for the observed differences in antibody responses. A decline in intestinal immunity may also reflect age-related decreases in enteric IgA produced in older children and adults, which may impact the overall neutralization potential of stool samples. While total IgA does decline with age^59^, it is readily detectable in stool from adult subjects and correction of polio type-specific responses to reflect total IgA concentration in stool has not been found to change the overall findings (Wright et al. unpublished data).

Likewise, it is possible that proteolytic degradation of IgA may occur during longer stool transit times in older individuals. However, experiments to evaluate the impact of prolonged incubation of neutralization-positive samples with stool from children and adults indicate that this is not a significant contributing factor (Wright et al. unpublished data). Decreased enteric IgA in older children and adults may also reflect physiologic changes that facilitate virus replication in localized areas of the intestine that are distinct from those typically associated with antigen-stimulated IgA secretion (e.g., Peyer’s patches). Alternatively, lower levels of total IgA in stool may stem from natural age-related involution of the adenoids, tonsils, and Peyer’s patches—all primary sites of poliovirus replication^60^.

## Induction and regulation of intestinal IgA responses to poliovirus

### Early development of IgA-mediated mucosal immunity in the intestine

The underlying question of why infants respond with robust mucosal antibody responses to OPV in the intestine, and why these responses wane with age remains unanswered and has clear implications for the global polio eradication effort. The assumption that IgA-mediated mucosal immunity is critical for control of enteric poliovirus replication is strongly reinforced by observations of the striking parallels between the sites of primary poliovirus replication in epithelial cells of the intestine, and in tissues associated with the mucosal immune system (e.g., adenoid, tonsil, and Peyer’s patches)^60^, and the same anatomic sites being responsible for induction and expression of mucosal IgA^61,62^.

While our current understanding of the maturation of the mucosal immunologic landscape in infants is incomplete, it is likely that factors associated with early immune regulation of polio-specific IgA in infancy play a significant role in determining the induction and maintenance of mucosal responses to the virus later in life. Indeed, administration of poliovirus vaccines during routine pediatric schedules generally coincides with a period of dynamic immunological development in infancy^63,64^. Production of endogenous IgA in the intestine commences during the first weeks of life and is associated with physiologic tissue maturation, immune activation, and the development of immunological tolerance to a wide range of orally ingested antigens^63,64^.

Importantly, microbial colonization of mucosal sites, which begins largely at birth, is a driving factor in shaping early innate and adaptive immune responses to both non-pathogenic and pathogenic organisms^65–69^. Immune responses to commensal microbes are directed toward establishing tolerance, limiting inflammation, and maintaining intestinal homeostasis^70,71^. However, early exposures to pathogenic microbes and antigens through infection or immunization concurrently shape the immunologic repertoire and establish critical effector memory responses^72^.

These responses are orchestrated within the highly adaptive microenvironment of the intestinal mucosa. Phagocytic macrophages and dendritic cells (DC), abundant in the lamina propria, continuously sample the luminal microbiota and effectively remove bacteria and pathogens that breach the epithelial barrier^73–75^. Microbe-laden DC are restricted to the mucosal compartment by mesenteric lymph nodes, providing a level of protection against systemic infiltration of pathogens into the body^76^.

The interplay of host-microbe mutualism is further refined to promote distinct adaptive immune responses in systemic and mucosal compartments. Systemic exposure to a range of microbial taxa leads to induction of a diverse antibody repertoire dominated by IgG and designed to prevent lethal microbial sepsis^77^. In contrast, mucosal exposure to microbiota elicits a more restricted response, primarily to microbial surface antigens and characterized by a more limited IgA repertoire, which rapidly declines in the absence of repeated antigenic exposure^77^. Moreover, while intestinal memory IgA responses increase after successive antigen exposures, they do not appear to show the synergistic increase typical of a systemic prime-boost response^78^.

This distinction is evident in the observed differences in antibody responses to systemic or orally delivered poliovirus vaccines in infants. Notably, subcutaneous or intramuscular injection of IPV elicits high-titered, serum IgG responses that are boosted by successive doses, but with little to no generation of intestinal IgA^11,17^. Conversely, OPV administered alone or in combination with IPV stimulates both serum and intestinal antibody responses, the latter dominated by enteric IgA. Recent studies demonstrate the influence of the intestinal microbiota on the magnitude of the enteric IgA antibody response to combined regimens of IPV and bOPV in infants, suggesting not only the presence but the composition of the microbiota shape the adaptive response to poliovirus in the intestine^79^.

Induction of mucosal IgA is further restricted by a higher required threshold for antigenic priming as compared to a systemic response^77^. In germ-free mice, transitory exposure to a non-replicating microbe within the intestine requires a dose several orders of magnitude higher than that of a systemic exposure to reshape the antibody repertoire^77^. It is possible then that failure to develop a strong intestinal IgA response to OPV challenge in older children and adults may reflect a sub-threshold level of antigenic exposure, due to significant microbial competition in the intestine or the transient nature of antigenic exposure conferred by a single challenge dose of live, attenuated OPV.

### Impact of environmental and host-related factors on intestinal immunity

Other environmental factors and host-related changes in enteric immunity may also play a role in modulating IgA responses to poliovirus vaccines in the intestine. Previous studies have noted impaired antibody responses to oral polio^25–27^ and rotavirus^80,81^ vaccines in developing country settings. Data on enteric immunity to poliovirus vaccines from several recent clinical trials is uniform across many countries^16–18^, although it does not reflect experience in some of the countries where OPV performance is diminished^28^. Parker and colleagues have postulated that interference with the induction of mucosal immunity to oral vaccines may be due to altered microbial populations in the gut, nutritional deficiencies or epigenetic factors that may preferentially affect children from low-income countries^82^. Impaired immunogenicity of OPV in children aged 6–11 months has been shown to correlate with recent infection with non-polio enteroviruses^83^. This observation supports a 2014 meta-analysis showing a consistent negative impact of concurrent infection with enteropathogens on OPV responses^84^, and underscores the potential impact of microbial inference in reducing induction of polio-specific IgA in the intestine.

### Regulation of mucosal IgA by immune cells in the intestine

Cell-mediated immunity to poliovirus in the intestine is much less well understood than antibody-mediated immunity in part, due to difficulties in obtaining adequate primary tissues and cells from this site in young children. Many of the detailed mechanisms of cell-mediated induction of IgA have been explored in animal models, and presented in comprehensive reviews^61,62,85–88^. While data from human studies is more limited, comparisons with murine models suggest the salient features of the gut mucosal immune system, and the functional outcomes needed to respond and adapt to luminal microbiota and protect against infection, are conserved between these species^89^.

In general, induction of antigen-specific IgA within the intestinal mucosa occurs in highly specialized germinal centers in Peyer’s patches and in isolated lymphoid follicles. Interaction of B cells with DC in the subepithelial dome of Peyer’s patches dictates the early stages of IgA induction^90^. Subsequent signals from CD4+ T follicular helper (TFH) and T regulatory (Treg) cells in the germinal centers of Peyer’s patches control B cell responses in an antigen-specific manner^91,92^, and provide signals that promote the switch to IgA-producing plasma cells^85,90^. IgA+ plasmablasts enter the circulation and subsequently home back to the intestine, where they take up residence in the lamina propria and constitute a primary effector arm of enteric immunity^91^.

Transport of antigen-specific IgA from the lamina propria through the epithelial layer occurs by interaction with polymeric Ig receptors (pIgR), which mediate transfer of IgA and help stabilize the structure of the secreted dimeric IgA molecule^93^. The rate of IgA transport across the mucosal epithelium is influenced in the lamina propria by Th17 cells that produce IL-17 in response to co-stimulation with cytokines^94–97^. Th17 cells contribute to maintaining intestinal homeostasis to commensal microbes via IL-17 induction of pIgR expression, thereby regulating the rate of IgA secretion into the lumen^98^.

### Cell-mediated suppression of vaccine-induced mucosal immunity

Maintenance of immune homeostasis in the intestine is critically dependent on constant adaptation of cell-mediated immune responses that regulate the quantity and specificity of secreted IgA^91,92,94^. It is puzzling then why older children and adults appear to lose the ability to mount a polio-specific IgA response in the intestine upon oral challenge with live poliovirus. The loss of detectable enteric IgA to OPV observed with increasing age raises questions, as to whether exposure to poliovirus vaccines in infancy may induce antigen-specific mucosal immune suppression or tolerance in the gastrointestinal tract^99^. As the gut is the primary site of exposure to the majority of foreign antigens early in life, mechanisms geared towards suppression of excessive immunologic reactivity and inflammation play a critical role in establishing and maintaining intestinal homeostasis^70^.

Several possible immunologic barriers may arise over time in the intestine to reduce or prevent IgA induction including inadequate cell-mediated support for IgA production or active suppression of enteric IgA induced by vaccine antigens. The latter concept was first demonstrated over 40 years ago in a series of studies showing that parenteral administration of cholera toxin in mice induced marked suppression of antigen-specific mucosal IgA in the intestine^100–103^. This suppressive effect could be adoptively transferred by a cellular fraction, presumed to be a suppressor T cell population^103^. Whether a similar phenomenon occurs in the context of parenteral administration of IPV in infancy remains a hypothesis to explain the lack of polio-specific enteric IgA responses observed in older children and adults with a history of childhood IPV.

Tregs with the potential for suppressor activity^104^ also play a critical role in IgA induction and maintenance of homeostasis to commensal microbes in the intestine^92,105–107^. In mice, depletion of CD4^+^ CD25^+^ Tregs significantly reduces intestinal IgA to microbial antigens, providing evidence of their essential role in regulating antibody responses to enteric microbiota^92^. At homeostasis, Tregs establish a tolerogenic state to commensal microbes by suppressing excessive inflammatory responses to microbial antigens and preventing gut pathology^107^.

However, in animal models and adult human studies, Tregs are also implicated in suppressing B cell responses to certain viral pathogens and vaccines^108–113^. In mice infected with Friend virus, Tregs suppress differentiation of germinal center B cells and plasma cells, and decrease B cell class switching through downregulation of CD68 expression, which reduces costimulation by TFH cells^112^. The suppressive effects of Tregs on immune responses to viral pathogens and vaccination are not universal, and distinct effects can be seen with different pathogens, at different stages of infection^111,114^ and after vaccination^113^, emphasizing the highly contextual nature of Treg suppressor activity.

Whether suppression of B cell immunity to poliovirus occurs in the intestine, and whether this is associated with the observed absence or loss of polio-specific enteric IgA in older children and adults is not known. In infants an abundance of Tregs home to the intestine in the first months of life, suggesting these cells play an essential role in shaping mucosal IgA responses to enteric microbiota^115,116^ Studies by Rabe and colleagues have shown that a higher proportion of circulating Tregs during the first 18 months of life is inversely related to the fraction of T cells that subsequently express a memory phenotype later in childhood^117^, suggesting these cells may further influence the long-term expression of immunologic memory to vaccine antigens encountered during infancy^117,118^.

### Modulation of poliovirus immunity in the intestine by vaccine adjuvants

Human Tregs and Th17 cells in the intestine display a high degree of plasticity^119,120^ and can be modulated by co-administration of vaccine antigens with certain adjuvants. Of interest, the enterotoxin-based mucosal vaccine antigen LT (R192G/L211A; dmLT) has been shown to induce strong antigen-specific Th17 responses and increase mucosal IgA to co-mixed antigens following parenteral or oral immunization^121–123^. Co-administration of dmLT with a dose-sparing amount of IPV in mice has been shown to promote polio-specific intestinal IgA and upregulate expression of the intestinal homing receptor α4β7 on trafficking T cells^122^. Clinical trials of dmLT co-administered with IPV in adults are ongoing and will yield valuable information on the impact of targeted modulation of cellular responses for induction of long-lived polio-specific mucosal IgA and virus neutralizing responses in the intestine.

### Contribution of tissue-resident memory T cells to poliovirus intestinal immunity

In the absence of detectable enteric IgA to poliovirus in older children and adults, control of poliovirus replication in the intestine may rely in part on tissue resident memory *T* (*T*_RM_) cell populations. *T*_RM_ are found in abundance in the epithelium of barrier sites such as the skin and mucosae, where they serve as sentinels to rapidly activate broad localized immunity^124–127^. *T*_RM_ play a central role in antiviral immunity through cytokine-mediated recruitment of both innate and adaptive immune cells, and by direct lysis of virally infected cells^128^. Unlike circulating CD8^+^ T-cells, *T*_RM_ in the gastrointestinal tract appear to be maintained independently of cognate antigen for long periods and may serve to limit the duration of virus shedding^129^. Whether these cells participate in controlling poliovirus replication in the intestine is not currently known. However, ongoing studies of intestinal immunity in adults with prior history of IPV or OPV in childhood will shed light on the impact of early exposure to poliovirus antigens on the development of long-term cell-mediated memory responses able to control virus shedding later in life.

## Conclusions

The impact of early childhood immunization with live and inactivated polio vaccines during a period of dynamic immunologic development is likely to play a significant role in establishing functional cell-mediated memory responses capable of stimulating mucosal IgA and restricting virus replication in the intestine. Current observations of an age-related decline in enteric mucosal IgA and virus neutralizing responses to OPV, associated with sustained virus shedding, raise important questions as to the nature of immune regulation of poliovirus replication in the intestine and have clear implications for global eradication efforts.

Promising new data is now emerging on the safety and immunogenicity of novel type 2 oral poliovirus vaccines (nOPV2) with improved genetic stability after intestinal passage^130,131^. These studies will undoubtedly further shape our understanding of the induction and regulation of mucosal immunity to poliovirus. With an increasing number of vaccine-derived poliovirus outbreaks occurring globally, deployment of nOPV2 and ongoing clinical development of nOPV types 1 and 3 will provide critical new tools to help mitigate the risk of further seeding of vaccine-derived virulent strains into vulnerable populations, and to reduce the number of cases of polio associated with these outbreaks.
